# Ultrasound-Guided Glenohumeral Joint Injection using a Modified Posterior Approach

**DOI:** 10.31138/mjr.200424.uig

**Published:** 2025-01-22

**Authors:** Evangelia E. Vassalou, Michail E. Klontzas, Matthaios Triantafyllou, Georgios A. Kakkos, Konstantinos Spanakis, Apostolos H. Karantanas

**Affiliations:** 1 Department of Medical Imaging, University Hospital of Heraklion, Heraklion, Crete, Greece; 2 Department of Radiology, School of Medicine, University of Crete, Voutes Campus, Heraklion, Greece

**Keywords:** glenohumeral joint, injection, intra-articular, ultrasound, approach, technique

## Abstract

Glenohumeral joint injections serve diagnostic and therapeutic purposes in a wide spectrum of shoulder disorders. Various techniques have been described, depending on the injection site, needle orientation and utilisation of image or landmark-based guidance. The accuracy of needle placement and clinical effectiveness vary by the injection route and the method of guidance and largely depend on operator’s expertise, clinical indication and patient’s profile. Herein, we describe an alternative ultrasound-guided glenohumeral joint injection technique through a modified posterior approach for routine use in clinical practice.

## INTRODUCTION

Injections into the glenohumeral joint (GHJ) represent a common clinical practice performed by a variety of health care providers including radiologists, orthopaedic surgeons, rheumatologists, and sports medicine specialists. Joint entry is primarily indicated for therapeutic intervention in adhesive capsulitis, degenerative joint disease and inflammatory arthritis, or for aspiration.^1,2^ Intraarticular contrast media administration is also essential for the conduction of arthrographic studies, improving the diagnostic performance of computed tomography (CT) and magnetic resonance imaging (MR). Finally, intraarticular lidocaine injection is indicated as an effective alternative to intravenous sedation for reducing anterior shoulder dislocations, particularly in the outpatient clinical setting or as a test for establishing the intraarticular source of pain.^3^

The GHJ injection technique may vary according to the utilised approach, performed through certain anatomical portals. In this context, the GHJ can be accessed either anteroinferiorly through the lower one-third of the joint, anterosuperiorly via the rotator interval or posteriorly via the posterior joint recess.^3^ The posterior anatomical portal is the most widely used and has been suggested to provide greater accuracy in GHJ injection and aspiration.^4^ However, the optimal injection portal and success rate of the various approaches, in terms of accurate needle placement and clinical effectiveness, remain controversial and largely depend on operator’s expertise, clinical indication, and patient’s body habitus.^5–7^

Interventions of the GHJ can be performed with image guidance, most commonly by the use of ultrasound (US) or fluoroscopy, or blindly by using palpation of anatomic landmarks. Image guidance is recommended to archive precision and avoid anatomical variations with numerous studies showing higher accuracy of image-guided over palpation-guided injections.^4,8^ In particular, US-guided are currently preferred over landmark-based or fluoroscopy-guided procedures as they have been reported to be more precise and cost-effective by enabling real-time visualisation of the needle while obviating radiation exposure.^9,10^

As US-guidance is gaining popularity and is recommended as the preferable means for GHJ injections, the purpose of this Technical Note is to introduce an alternative US-guided GHJ injection technique through a modified posterior approach for routine use in clinical practice. Detailed technical considerations for enabling reproducibility together with description of the specific advantages of the proposed method are provided.

## US-GUIDED INJECTION TECHNIQUE

### Equipment

The procedure should be performed with a high-resolution (6–15 MHz) linear-array transducer. The use of sterile probe cover, sterile gloves, fenestrated drape and sterile ultrasound gel is suggested. Utilisation of a 21-gauge (0.8 mm) needle of 3.8 cm in length is adequate for the intraarticular injection. The type of syringe and injected medication depend on the clinical indication.

### Patient’s positioning and skin preparation

**Figure 1.**
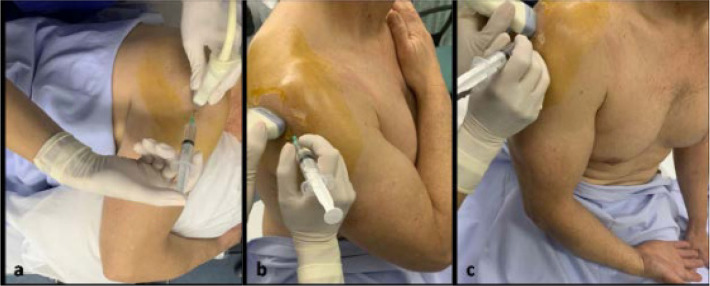
Patient’s positioning options for performing the modified posterior glenohumeral joint injection. The patient is placed in the semiprone position with the elbow flexed at 90^o^ and the forearm hanging over a pillow (**a**) or in the seated position with the hand either on the contralateral shoulder (**b**) or resting on the contralateral thigh in supination (**c**).

Before the procedure ordinary skin antisepsis should be applied over the posterior and lateral aspect of the shoulder. The patient could be placed either: (i) in the semiprone position with the ipsilateral to the injected side elbow flexed at 90^o^ and the forearm placed over a pillow to maximise comfort, or (ii) in the seated position with the hand on the injected side positioned on the contralateral shoulder or resting on the contralateral thigh with the forearm in supination (**Figure 1**). The semiprone body position is preferred by the authors as it enables easy access to the whole periphery of the shoulder girdle, ensures patient’s stability and eliminates potential consequences related to vagal reactions (**Supplementary Video 1**).

### Scanning technique and injection

**Figure 2.**
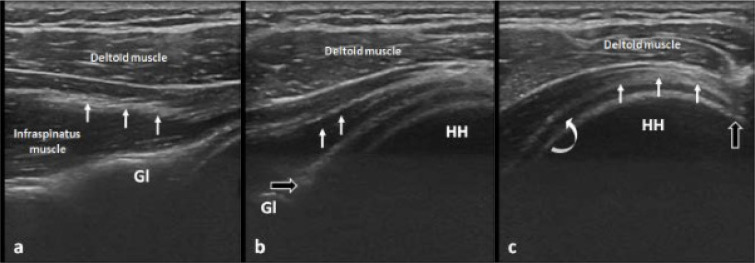
(**a**) Sonographic image along the long axis of the infraspinatus muscle obtained by placing the probe just inferior and parallel to the scapular spine, showing the infraspinatus central tendon (arrows) lying superficial to the glenoid (Gl). (**b**) Sonographic image obtained by moving the transducer lateral to “a” shows the infraspinatus myotendinous junction (white arrows) at the level of the posterior joint recess overlying the posterior labrum (black arrow), glenoid (Gl) and medial part of the humeral head (HH). (**c**) Sonographic image obtained by moving the transducer lateral to “b” shows the infraspinatus tendon (arrows) overlying the articular cartilage (curved arrow) of the humeral head (HH) and inserting on the greater tuberosity (black arrow).

**Figure 3.**
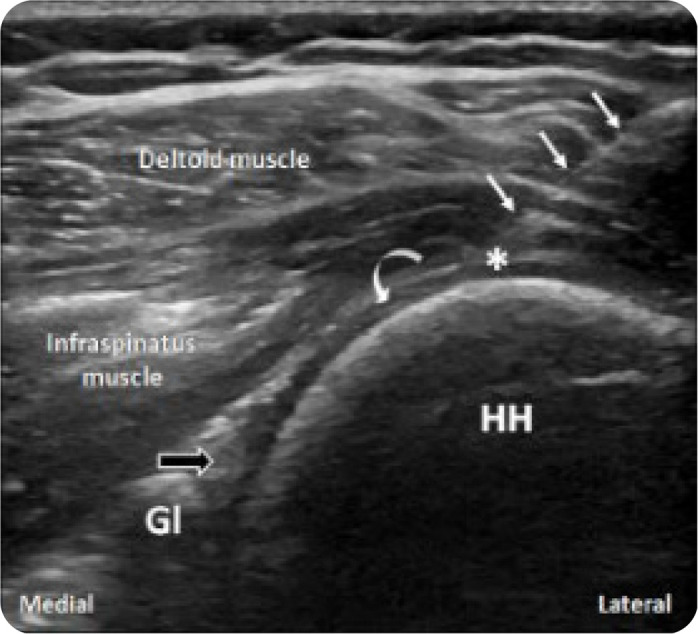
Sonographic image along the long axis of the infraspinatus tendon showing needle advancement (thin arrows) to the target point (*) immediately superficial to the articular cartilage (curved arrow) of the humeral head (HH). The glenoid (Gl) and the posterior labrum (black arrow) can be seen at the level of the posterior joint recess.

**Figure 4.**
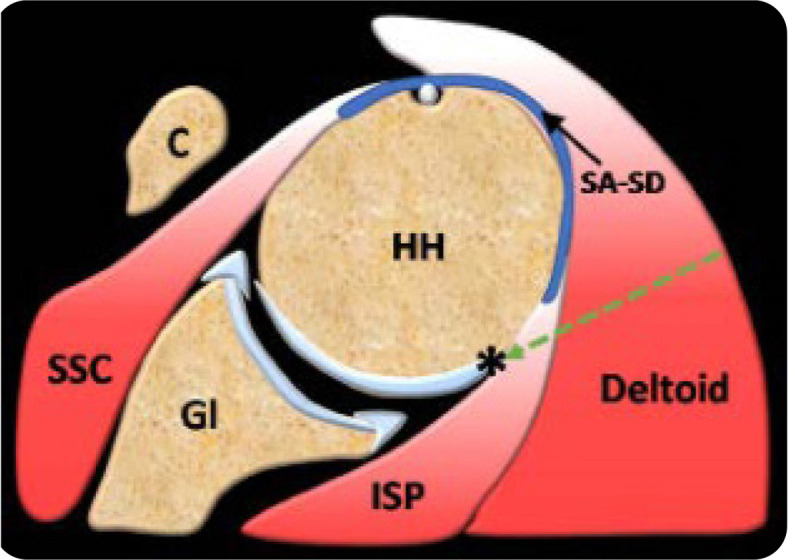
Schematic drawing showing the needle route (green arrow) and target point (*) during the modified posterior approach. HH: humeral head; Gl: glenoid; C: coracoid process; SSC: subscapularis muscle and tendon; ISP: infraspinatus muscle and tendon; SA-SD: subacromial-subdeltoid bursa.

The probe should be initially placed parallel and just inferior to the scapular spine, enabling visualisation of the infraspinatus muscle with its central tendon, lying deep to the deltoid muscle and superficial to the glenoid. The probe should be then moved laterally allowing depiction of the infraspinatus myotendinous junction coursing deep to the deltoid muscle and superficial to the posterior labrum and glenoid, and medial part of the humeral head. The probe should be moved further laterally while maintaining alignment with the long axis of the infraspinatus tendon to the point where the latter can be seen inserting on the posterior greater tuberosity at the posterolateral aspect of the humeral head (**Figure 2**). The injection spot is located 2 cm medial to the infraspinatus tendon insertion, just superficial to the hypoechogenic articular cartilage overlying the humeral head (**Figure 3**). Once the target has been defined, the needle is introduced at the skin surface approximately 2 cm lateral to the lateral heel of the transducer at a 45^o^ angle, allowing a shallow needle trajectory and maximal needle visibility. The needle is advanced in an oblique lateral-to-medial direction through an in-plane approach towards the humeral head, under real-time sonographic guidance. A schematic drawing showing the needle route and target point is provided in **Figure 4**. The soft and spongy feeling of the articular cartilage confirms that the needle tip has reached the target point and the injection can be initiated (**Supplementary Videos 2 and 3**). The injectate should be dispersed intraarticularly with mild or no resistance. In case of difficulty in injecting the agent, the needle should be slightly retracted as to ensure non-contact with the articular cartilage and needle bevel obstruction. Constant US monitoring is essential for avoiding inadvertent extraarticular injection or accumulation of the injectate into untargeted structures.

## DISCUSSION

Depending on the injection portal, needle orientation, and utilisation of US or fluoroscopic image guidance, various GHJ injection techniques have been proposed. Although injection through the posterior portal and utilisation of US guidance have been reported to be more effective than alternative methods, there is still controversy regarding the ideal injection technique in terms of accuracy and clinical effectiveness.^5,6^

Herein we describe an alternative US-guided method for injecting the GHJ through a modified posterior approach. The proposed technique requires a short-length needle, enables a short injection path and avoids needle advancement through the anterior and posterior stabilising structures and the infraspinatus muscle, besides does not entail any risk for labral or neurovascular injury.

Compared to the anteroinferior approach through the lower one-third of the GHJ joint, the modified posterior injection technique does not affect the anatomy of this certain anatomical site. This is of clinical importance especially when GHJ injection is performed in the context of MR arthrography for the assessment of the anterior labroligamentous complex or capsule. As the needle entry through the anteroinferior part of the joint may distort the anterior stabilising structures or cause extravasation through the injection path, it may cause diagnostic dilemmas on the interpretation of the arthrographic study.^11^ Additionally, the proposed modified posterior portal is not associated with risk of axillary neurovascular bundle or brachial plexus injury, while occurrence of these complications has been reported during the anteroinferior approach.^11^

A potential pitfall related to anterosuperior GHJ insertion through the rotator cuff interval, which could be avoided by using the modified posterior approach, is unintentional injection of the subacromial-subdeltoid bursa due to shallow needle placement, considering their anatomical proximity, with the bursa overlying the rotator interval.^11^ This has significant clinical impact, as accidental bursal injection during MR or CT arthrography may lead to a false-positive diagnosis of full-thickness rotator cuff tear.^12^ Another limitation of the anterosuperior approach, which could be precluded with the modified posterior technique, is potential accumulation of contrast material within the rotator cuff interval possibly obscuring abnormalities located in this anatomical area.^11^

Different to the modified posterior GHJ approach, the classical posterior portal carries the risk of injury to neurovascular bundles, mainly the suprascapular nerve and circumflex scapular vessels, or the posterior glenoid labrum if the needle is directed far too medially, especially in massive shoulders where a low-frequency probe may be necessary for optimal visualisation of the deep structures.^13^ Additionally, the modified posterior approach enables a more direct and shorter needle path compared to the posterior entry. This is of clinical importance as a 3.8-cm needle length is adequate for the modified posterior, compared to a 7.5–10 cm needle which is often required to reach the necessary depth when injecting through the classical posterior approach. Besides, during needle advancement through the infraspinatus muscle and the posterior GHJ recess, the needle may be deflected off course.^14^ The shorter needle path when injecting via the modified posterior approach, not requiring needle advancement through the infraspinatus muscle, aids in avoiding needle disorientation.

Due to the required close proximity with the needle bevel, injury to the articular cartilage overlying the humeral head is a potential risk related to the modified posterior joint entry. However, avoiding further needle advancement once the characteristic spongy feeling of the articular cartilage is confirmed could prevent this procedure-related complication.

In conclusion, a novel technique for GHJ injection via a US-guided modified posterior approach is described herein. The standardised protocol and the US anatomical landmarks are provided for ensuring reproducibility, together with description of the specific advantages of the method. Further research focused on the feasibility, learning curve and effectiveness, in terms of accurate needle placement and clinical outcome, would allow for a comparative analysis between the modified posterior and alternative GHJ injection approaches.

## FUNDING

This research did not receive any specific grant from funding agencies in the public, commercial, or not-for-profit sectors.

## CONFLICT OF INTEREST

The authors declare no conflict of interest.

## Figures and Tables

**Supplementary Video 1 (left).** Video showing the real-time patient’s preparation for performing the modified posterior glenohumeral joint approach.

**Supplementary Video 2 (below).** Real-time sonographic imaging along the long axis of the infraspinatus tendon and posterior glenohumeral joint recess showing in-plane needle advancement in an oblique lateral-to-medial direction through the infraspinatus tendon towards and just superficial to the articular cartilage of the humeral head, with the target point located about 2 cm medial to the insertion of the infraspinatus tendon on the greater tuberosity.

**Supplementary Video 3.** Real-time sonographic imaging along the long axis of the infraspinatus tendon and posterior glenohumeral joint recess following needle advancement just superficial to the humeral head articular cartilage via the modified posterior approach shows unrestricted intraarticular accumulation of the injectate within the posterior joint recess.
